# Venous thromboembolism, can we do better? Profile of venous thromboembolism risk and prophylaxis in a University Hospital in the State of São Paulo

**DOI:** 10.1590/1677-5449.004018

**Published:** 2019-02-27

**Authors:** Arthur Curtarelli, Luiz Paulo Correia e Silva, Paula Angeleli Bueno de Camargo, Rafael Elias Farres Pimenta, Rodrigo Gibin Jaldin, Matheus Bertanha, Marcone Lima Sobreira, Winston Bonetti Yoshida

**Affiliations:** 1 Universidade Estadual “Júlio de Mesquita Filho” – UNESP, Faculdade de Medicina de Botucatu (FMB), Hospital das Clínicas (HC), Botucatu, SP, Brasil.

**Keywords:** venous thromboembolism, venous thrombosis, emboli, thrombosis

## Abstract

**Background:**

Venous thromboembolism (VTE) is a silent and potentially lethal disease that affects a considerable proportion of hospitalized patients. It has high morbidity and mortality and is responsible for a heavy financial burden on healthcare systems. However, VTE can be prevented using prophylaxis measures that have been established in the literature. Nonetheless, in the real world, mean rates of appropriately administered VTE prophylaxis are lower than 50%.

**Objectives:**

To define the epidemiological profile of patients with VTE in a University Hospital and the rate of appropriately administered VTE prophylaxis at that service and to identify measures to improve the rate.

**Methods:**

A cross-sectional, observational study was conducted with data collected from the medical records of patients who met the inclusion criteria. The rates of correct VTE prophylaxis prescribed to clinical and surgical patients were compared, assessed according to guidelines published by the Brazilian Society of Angiology and Vascular Surgery (SBACV), based on VTE risk classification.

**Results:**

The overall rate of correctly-prescribed VTE prophylaxis was 42.1%, while 57.9% of patients were not managed correctly in this respect. Clinical patients had a 52.9% rate of appropriate prophylaxis, while the equivalent rate for surgical patients was 37.5%.

**Conclusions:**

Rates of correctly-prescribed VTE prophylaxis are still lower than they should be. Ongoing education, measures to encourage bedside risk stratification, and improvements to the electronic prescription system could increase appropriate VTE prophylaxis rates.

## INTRODUCTION

 Pharmaceutical and mechanical prophylactic measures to prevent venous thromboembolism (VTE) are well-established in international consensuses, for both clinical and surgical patients, 1 ^,^ 2 based on risk stratification models. 3 ^-^ 5 However, many Brazilian 5 ^,^ 6 and international publications show that, in the real world, approximately 50% of patients at risk of VTE are not being prescribed chemical prophylaxis when it is indicated, or are being given inappropriate prophylaxis. 7 ^,^ 8 Rates of correctly-prescribed prophylaxis vary across different countries and different services from 2 to 92%. 9 ^-^ 11 According to the ENDORSE study, Brazil has inappropriate prophylaxis rates of 41% for clinical patients and 54% for surgical patients. 11 Some Brazilian studies have found even higher rates of up to 61% for clinical and surgical patients. 12

 In addition to the morbidity and mortality that a hospital stay complicated by VTE can cause (2 million cases of deep venous thrombosis (DVT) and 200 thousand deaths/year in the United States, for example), the financial costs of the disease are also a cause for concern among administrators and managers. In one University Hospital in Brazil, the in-hospital VTE treatment cost, to the point at which therapeutic levels are achieved, varied from US$ 69.11 when treated with low molecular weight heparin (LMWH) to US$ 88.39, when unfractionated heparin (UFH) was used, covering only the costs of the materials and medications employed, excluding the infusion pumps. 13 Much higher sums are observed when the full treatment provided for this disease over a 90-day period with home care is considered: a retrospective cohort study in Canada reported figures of US$ 9,347.00 when treated with LMWH and US$ 11,930.00 when treated with UFH. 14

 According to Brazilian and international guidelines, introduction of Hospital VTE Prevention Commissions (HVTEPC) would be an important element for improving VTE prevention. 15 ^-^ 17 Although VTE prophylaxis is well-established in Brazilian and international consensuses, it is disconcerting that it is still not being appropriately administered at Brazilian health services. 

 São Paulo’s state universities run the largest and most important university hospitals outside of the state capital and are responsible for providing care to a significant proportion of the population that is dependent on the Brazilian National Health Service (SUS - Sistema Único de Saúde). Implementation of HVTEPC is being rolled out timidly at these institutions and it is necessary to conduct a wide-ranging survey of the true state of VTE prophylaxis. 

 The objectives of this study were to assess the risk profile of patients admitted to a public university hospital in Brazil, determine rates of inappropriate VTE prophylaxis, identify the causes of these failures, and suggest measures to solve the problem. 

## METHODS

 This is a cross-sectional, observational study investigating adult patients over the age of 18 years admitted to a public university hospital in Brazil and treated by the SUS, from October 2015 to February 2016, by orthopedic surgery, general surgery, gastrointestinal surgery, vascular surgery, urology, gynecology, internal medicine, and intensive care specialties. Patients already assessed during previous admissions were not reassessed and pregnant women, patients with contraindications to anticoagulants, with indications for vena cava filters, on full anticoagulation, or not meeting the inclusion criteria outlined above were all excluded. 

 Data were collected from information on the electronic medical records of patients admitted to the hospital using MV-PEP® software, with prior authorization from the hospital’s Research Ethics Committee and consent from the physician responsible for each specialty, but without informing the treating teams in advance. 

 The sample size was defined after a statistical assessment using preliminary data from a pilot study with information on 80 patients. The sample size was estimated at 500 patients, with similar numbers of patients from each specialty. Data were collected at random as patients were admitted during the data collection period. 

**Figure 1:**
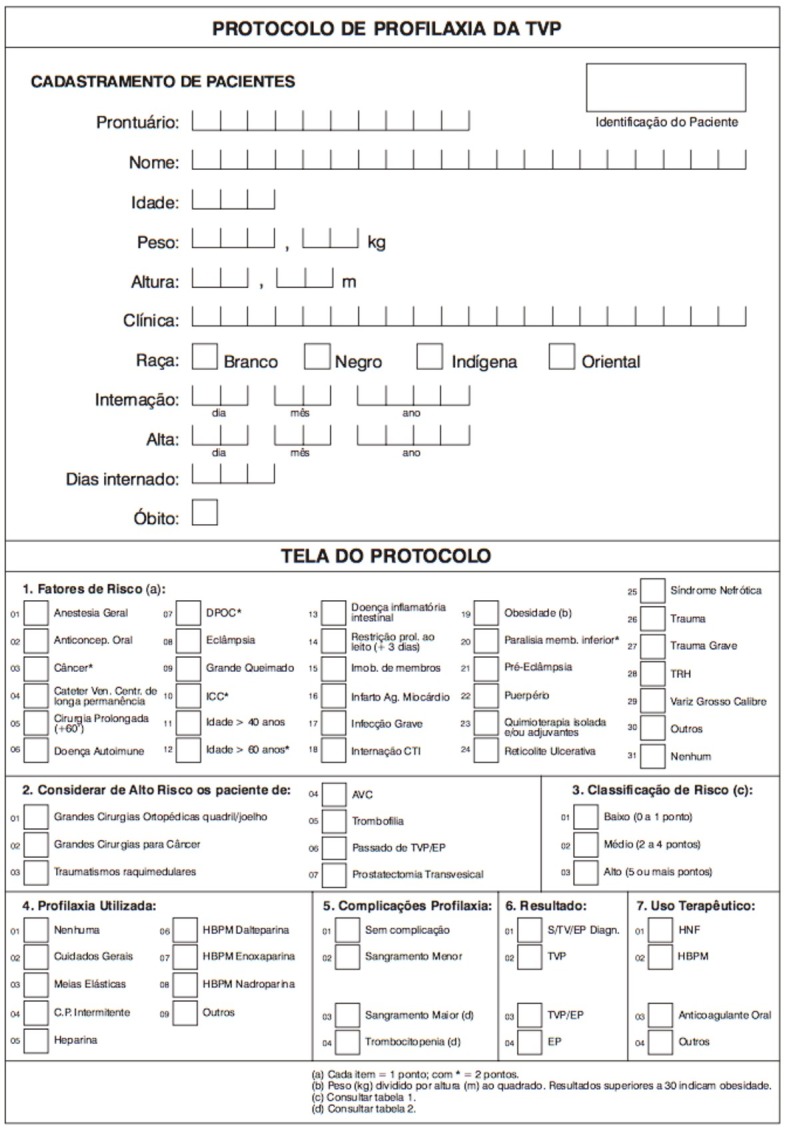
Brazilian Society of Angiology and Vascular Surgery deep venous thrombosis prophylaxis protocol (in Portuguese).

 The 2005 VTE prophylaxis guidelines published by the Brazilian Society of Angiology and Vascular Surgery (SBACV) were used to determine risk and prophylaxis indications ( Figure 1 ). Data were collected by the researchers and tabulated in an Excel® spreadsheet in a standardized manner for later statistical analysis. 

 Patients were separated into two major groups (clinical and surgical) and then subdivided according to SBAVC VTE risk strata into low and high risk clinical patients and low, moderate, and high risk surgical patients risk. 17 Results were then analyzed to determine the relationship between risk classification and prophylaxis prescribed, defining prophylaxis as appropriate only if it complied with the criteria set out in the SBACV VTE guidelines. Data and results were double-checked by the researchers. 

 Only mechanical prophylaxis measures that could be selected on the hospital’s MV-PEP® system were defined as correct, as follows: instruct/encourage early mobilization and motor physiotherapy. Other methods of mechanical prophylaxis, such as graduated compression elastic stockings and/or intermittent pneumatic compression devices were not available to the healthcare team. Along the same lines, the only pharmaceutical prophylaxis measures considered appropriate were those set out in the SBACV guidelines and available on the hospital’s MV-PEP® system: 20 mg enoxaparin once a day, 40 mg enoxaparin once a day, 5,000 international units (IU) of unfractionated heparin every 8 hours or every 12 hours, and 2.5 mg fondaparinux once a day. Dalteparin and nadroparin, which are also recommended by the SBACV, were not available at this hospital. 

 Statistical analysis was performed using the Statistical Package for the Social Sciences (SPSS®), using the chi-square test for categorical variables and Student’s *t* test to compare the means of continuous variables, with statistical help provided by the institution’s research support office. 

## RESULTS

### Eligibility and demographics

**Figure 2:**
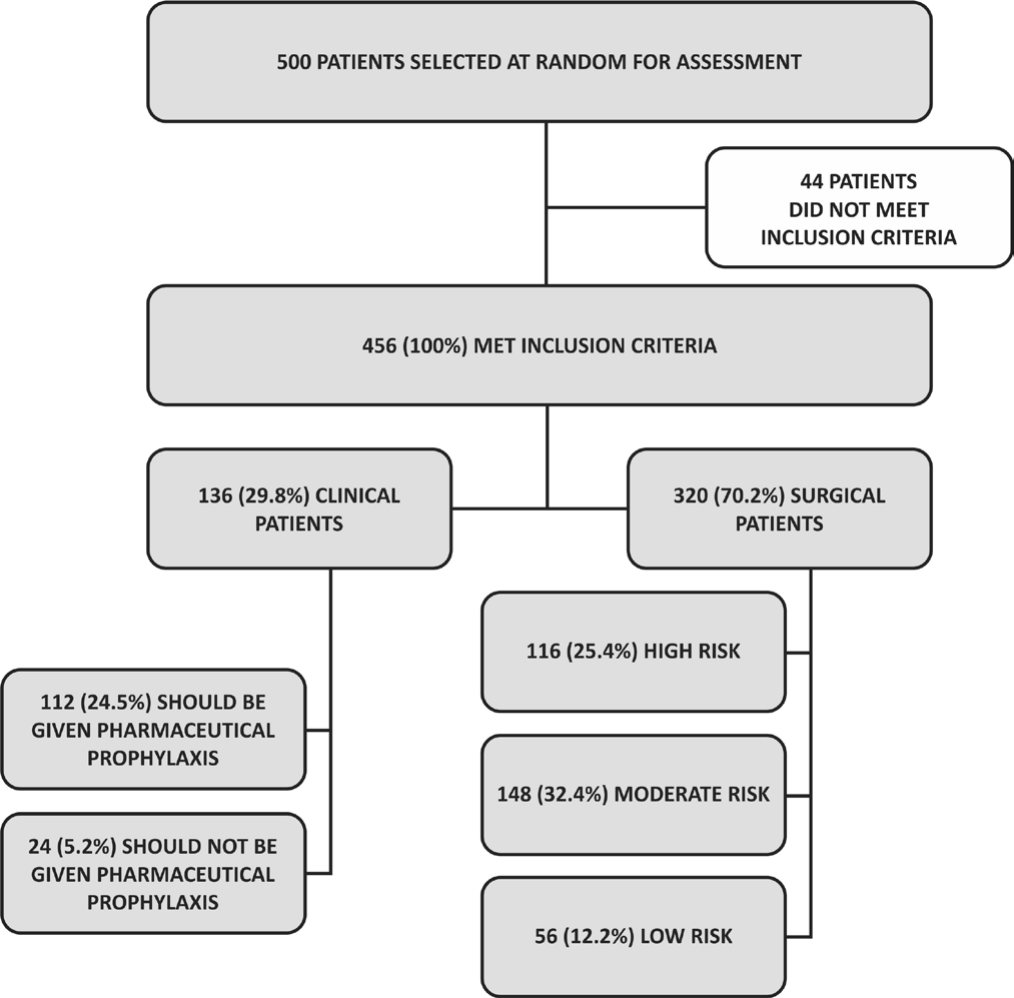
Flow diagram showing inclusions and exclusions and risk of venous thromboembolism.

 Electronic patient records were analyzed for 500 patients selected at random, 456 (100%) of whom met the inclusion criteria. The sample was divided into two categories: A) clinical patients (n = 136, 29.8%) and B) surgical patients (n = 320, 70.2%), on the basis of the protocols for risk and prophylaxis indications produced by the SBACV. The 44 patients were excluded from the sample for the following reasons: eight had indications for vena cava filters, 16 were pregnant women, and 20 were on full anticoagulation ( Figure 2 ). 

 The patients analyzed were hospitalized for a variety of reasons and for periods judged necessary by the healthcare team, which was responsible for choosing VTE prophylaxis prescriptions. 

 Male patients predominated in both the clinical (52.6% men vs. 47.4% women) and the surgical groups (60.6% men vs. 39.4% women). The clinical group had higher mean age than the surgical group by 7.4 years (62.4 vs. 55.0 years, respectively) and its mean length of hospital stay was 1.5 days longer than in the surgical patient group (6.1 days vs. 4.6 days). Table 1 illustrates the demographic profile of the patients. 

**Table 1:** Risk factors for VTE by patient group, clinical or surgical.

**Risk factor**	**Clinical (%)**	**Surgical (%)**
Mean age	62.4 years	55.0 years
Length of hospital stay	6.1 days	4.6 days
Age > 60 years	59.2%	47.7%
Sepsis/ severe infection	57.9%	16.1%
Sex (female/male)	47.4%/52.6%	39.4%/60.6%
Admitted to ICU	4.6%	6.9%
Cancer surgery	3.9%	8.0%
Duration of surgery > 60 minutes	3.9%	38.8%
AMI	3.9%	1.4%
Nephrotic syndrome	3.3%	0.3%
Age range: 40-60 years	28.3%	28.7%
Cancer	23.7%	14.1%
Confined to bed > 3 days	23.7%	13.7%
CHF	20.4%	6.3%
Oral contraception	2%	0.6%
Lower limb paralysis	2.0%	1.1%
Stroke	2.6%	0.0%
General anesthesia	2.6%	24.1%
Intense varicose veins	2.6%	5.5%
Long stay venous catheter	14.5%	12.1%
Prior history of PTE/DVT	12.5%	6.6%
Chemotherapy	11.8%	3.4%
COPD	11.2%	3.4%
Autoimmune disease	1.3%	1.4%
Trauma	1.3%	19.0%
Thrombophilia	0.7%	0.0%
Transvesical prostatectomy	0.7%	1.4%
Orthopedic hip or knee surgery	0.0%	4.0%
Spinal cord trauma	0.0%	9.0%
Extensive burns	0.0%	0.5%
Immobilization of limbs	0.0%	21.3%
Ulcerative rectocolitis	0.0%	0.6%
Hormone replacement therapy	0.0%	0.0%

PTE = pulmonary thromboembolism, DVT = deep venous thrombosis, COPD= chronic obstructive pulmonary disease, CHF = chronic heart failure, AMI = acute myocardial infarction, ICU = intensive care unit.

### Risk factors

 In common with the demographic profile, risk factors were different in the two both groups. There were more risk factors related to chronic diseases (cancer, chronic obstructive pulmonary disease, congestive heart failure, acute myocardial infarction, bed confinement, and prior history of VTE) and chemotherapy medication in the clinical group, whereas factors predisposing to VTE in the surgical group were related to the surgical intervention itself (trauma, tissue wounds, general anesthesia, and postoperative immobilization) ( Table 1 ). 

### Overall outcomes (clinical and surgical patients)

 The overall rate of appropriate VTE prophylaxis prescriptions was 42.1%, vs. 57.9% inappropriate VTE prophylaxis, for the whole sample of 456 patients analyzed ( Table 2 ). 

**Table 2:** Overall analysis of appropriate and inappropriate prophylaxis (p < 0.05).

	**Clinical** **n (%)**	**Surgical** **n (%)**	**% total**
Total	136 (29.8%)	320 (70.2%)	100%
Appropriate prophylaxis	72 (15.7%)	120 (26.3%)	42.1%
Inappropriate prophylaxis	64 (14.1%)	200 (43.8%)	57.9%

**Figure 3:**
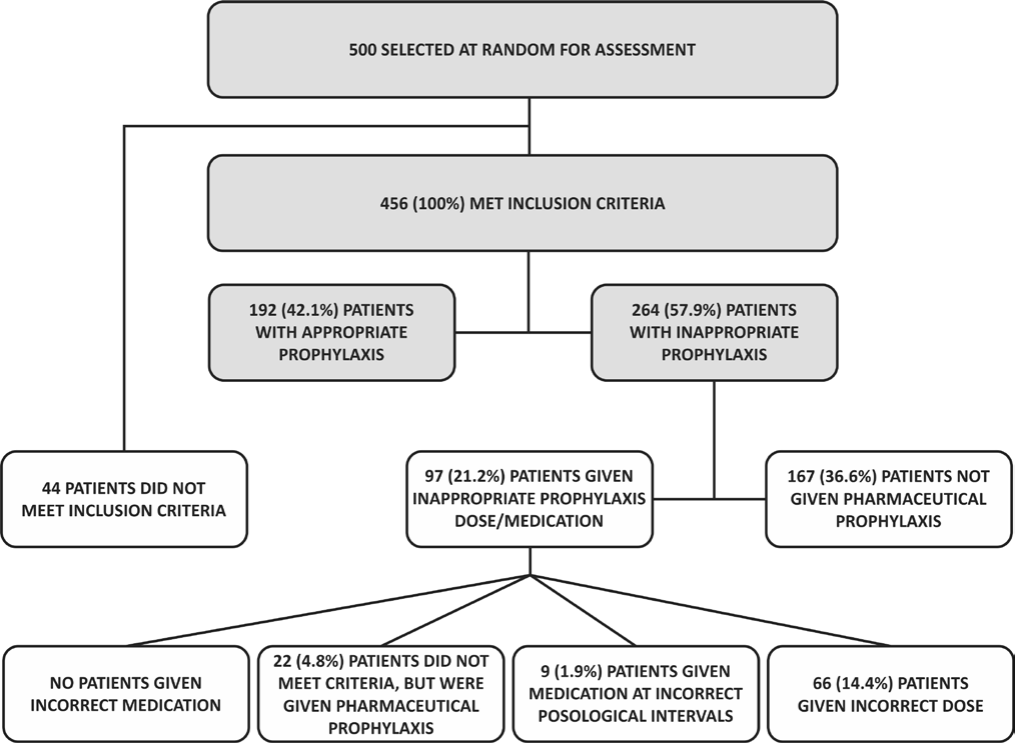
Flow diagram showing inclusions and exclusions, appropriate prophylaxis, and inappropriate prophylaxis with subtypes for all patients.

 Analysis of the 57.9% (264 patients) with inappropriate VTE prophylaxis revealed that 36.6% (167 patients) met criteria for pharmaceutical prophylaxis, but were not given it; 14.4% (66 patients) were given it, but at the wrong dosage; 1.9% (nine patients) were given it but with incorrect dose intervals; and 4.8% (22 patients) were given medication despite not meeting criteria for pharmaceutical prophylaxis ( Table 3 and Figure 3 ). 

**Table 3:** Stratified analysis of types of inappropriate prophylaxis for all patients with inappropriate prophylaxis (p < 0.05).

**Patients with inappropriate prophylaxis**		**overall n (%)**	**overall n (%)**
Not given pharmaceutical prophylaxis			167 (36.6%)
Given inappropriate prophylaxis	Given incorrect dose	66 (14.4%)	97 (21.2%)
	Administered at incorrect posological intervals	9 (1.9%)	
	Did not meet criteria, but were given pharmaceutical prophylaxis	22 (4.8%)	
	Given incorrect medication	0 (0.0%)	

### Outcomes in the clinical group

 Analysis of the 136 (100%) clinical patients in isolation revealed that 72 (52.9%) of them were given pharmaceutical prophylaxis correctly, in conformity with the SBACV guidelines, while 64 (47.1%) patients had pharmaceutical prophylaxis managed incorrectly ( Table 4 ). 

**Table 4:** Analysis of appropriate and inappropriate prophylaxis for all clinical patients.

**Clinical patients**	**n total**	**% total**
Appropriate prophylaxis	72	52.9
Inappropriate prophylaxis	64	47.1

 The SBACV classifies clinical patients into two groups, those at increased risk, who should be given pharmaceutical prophylaxis, and those at lower risk, who should not be given prophylactic medications and should be treated with mechanical prophylaxis only. The clinical patients were therefore classified according to the SBACV guidelines and analyzed separately for correct or incorrect pharmaceutical prophylaxis. 

 There were a total of 112 clinical patients at increased risk, among whom prophylaxis was prescribed correctly (i.e., pharmaceutical prophylaxis was given) for 60 (44.1% of the clinical patients) and incorrectly for the remainder (38.2% of the clinical patients) ( Table 5 ). 

**Table 5:** Analysis of clinical patients stratified by need for prophylaxis and appropriate or inappropriate prophylaxis (p < 0.05).

**Risk of DVT/PTE**	**Clinical patients** **n (%)**	**Inappropriate prophylaxis** **n (%)**	**Appropriate prophylaxis** **n (%)**
Should be given pharmaceutical prophylaxis	112 (82.3%)	52 (38.2%)	60 (44.1%)
Should not be given pharmaceutical prophylaxis	24 (17.7%)	12 (8.8%)	12 (8.8%)

**Figure 4:**
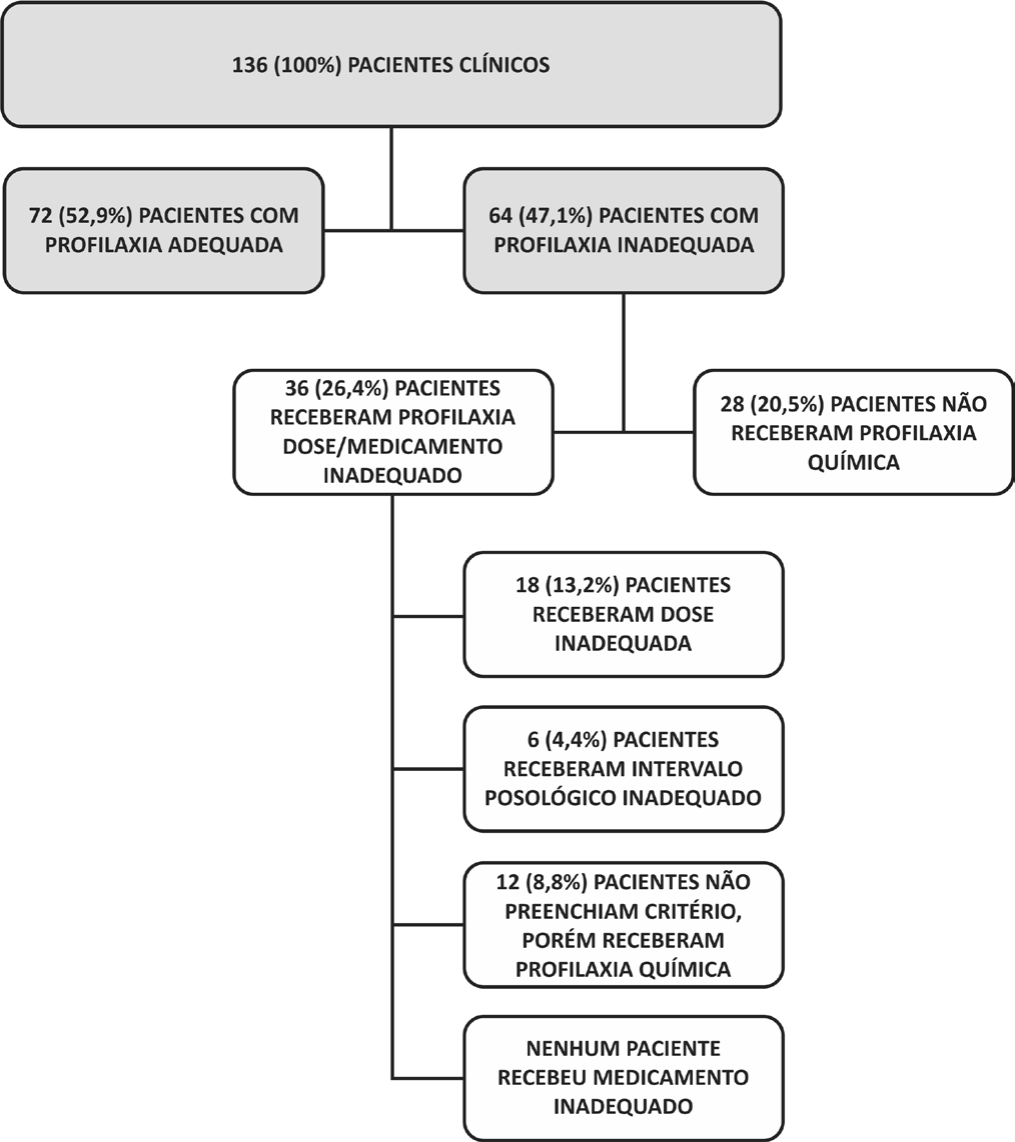
Flow diagram showing inclusions and exclusions, appropriate prophylaxis, and inappropriate prophylaxis with subtypes for clinical patients.

 Analyzing just those clinical patients for whom pharmaceutical prophylaxis was managed incorrectly (64 patients) we observed that 28 (20.5% of the clinical patients) patients were not given medication despite meeting the criteria, whereas the other 36 (26.4% of the clinical patients) patients were prescribed pharmaceutical prophylaxis, but not in accordance with the SBACV protocol, because dosages or frequency were incorrect, or were given medication when they did not meet criteria for pharmaceutical prophylaxis ( Table 6 and Figure 4 ). 

**Table 6:** Stratified analysis of types of inappropriate prophylaxis for clinical patients with inappropriate prophylaxis (p < 0.05).

**Clinical patients with inappropriate prophylaxis**		**overall n (%)**	**overall n (%)**	**% of patients with inappropriate management**
Not given pharmaceutical prophylaxis			28 (20.5%)	43.8%
Given inappropriate prophylaxis	Given incorrect dose	18 (13.2%)	36 (26.4%)	56.2%
	Administered at incorrect posological intervals	6 (4.4%)		
	Did not meet criteria, but were given pharmaceutical prophylaxis	12 (8.8%)		
	Given incorrect medication	0 (0.0%)		

### Outcomes in the surgical group

 Analysis of the group of surgical patients, comprising 320 (100%) individuals, found a 37.5% (120 patients) rate of appropriate prophylaxis and a 62.5% (200 patients) rate of noncompliance with the SBACV guidelines ( Table 7 ). 

**Table 7:** Analysis of appropriate and inappropriate prophylaxis for all surgical patients.

**Surgical patients**	**n total**	**% total**
Appropriate prophylaxis	120	37.5
Inappropriate prophylaxis	200	62.5

 The SBACV guidelines have three categories for surgical patients: low, moderate, and high risk, each of which has its own appropriate measures for pharmaceutical prophylaxis. 

 It was observed that 37.5% of surgical patients were given the appropriate prophylaxis, distributed by risk category as follows: 14.4% were at low risk of DVT/PTE, 5.3% were at moderate risk, and 17.8% were at high risk ( Table 8 ). 

**Table 8:** Analysis of surgical patients stratified by risk classification and appropriate or inappropriate VTE prophylaxis (p < 0.05).

**Risk of DVT/PTE**	**Surgical patients** **n (%)**	**Inappropriate prophylaxis** **n (%)**	**Appropriate prophylaxis** **n (%)**
High risk	116 (36.2%)	59 (18.4%)	57 (17.8%)
Moderate risk	148 (46.2%)	131 (40.9%)	17 (5.3%)
Low risk	56 (17.5%)	10 (3.1%)	46 (14.4%)

**Figure 5:**
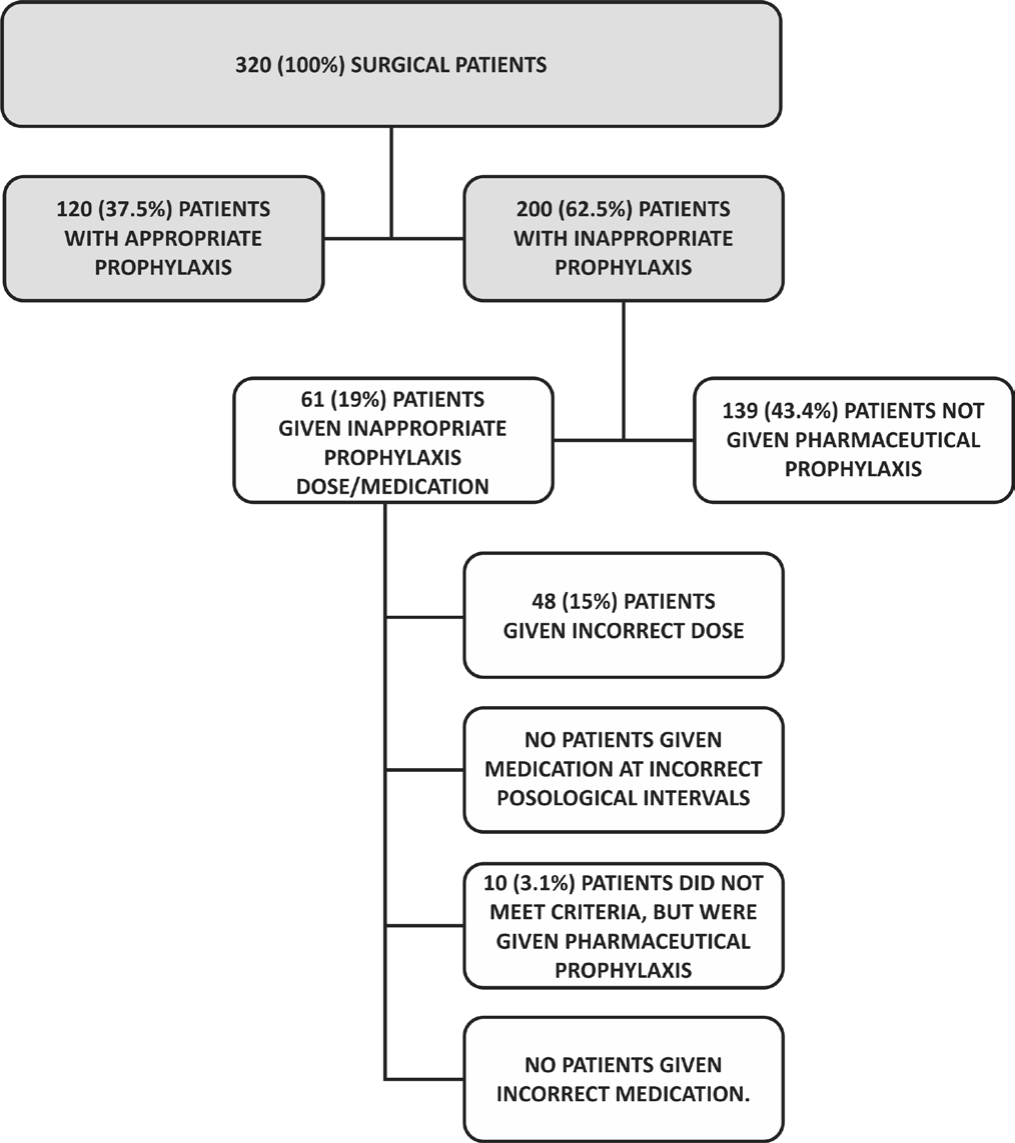
Flow diagram showing inclusions and exclusions, appropriate prophylaxis, and inappropriate prophylaxis with subtypes for surgical patients.

 Of the 200 patients put on a prophylactic regime different from that recommended by SBACV guidelines, 139 (43.4%) patients were not given pharmaceutical prophylaxis even though they met criteria for medication, while 61 (19.0%) patients were given pharmaceutical prophylaxis, but with incorrect dosage or posology, or were given medication despite not meeting criteria for pharmaceutical prophylaxis ( Table 9 and Figure 5 ). 

**Table 9:** Stratified analysis of types of inappropriate prophylaxis among surgical patients with inappropriate prophylaxis (p < 0.05).

**Surgical patients with inappropriate prophylaxis**		**overall n (%)**	**overall n (%)**	**overall n (%)**	**overall n (%)**
Not given pharmaceutical prophylaxis					139 (43.4%)
Given inappropriate prophylaxis		Low risk	Moderate risk	High risk	61 (19.0%)
	Given incorrect dose	0 (0.0%)	32 (10.0%)	16 (5.0%)	
	Administered at incorrect posological intervals	0 (0.0%)	0 (0.0%)	0 (0.0%)	
	Did not meet criteria, but were given pharmaceutical prophylaxis	10 (3.1%)	0 (0.0%)	0 (0.0%)	
	Given incorrect medication	0 (0.0%)	0 (0.0%)	0 (0.0%)	

## DISCUSSION

 The data collected reiterate the epidemiological profile described in the literature on patients at increased risk of VTE, since we found that patients had multiple comorbidities, age greater than 60 years, and mean length of hospital stay in the range 4.6 to 6.1 days. Surgical patients appear to be at greater risk, because of factors directly related to their surgical procedures. 

 Considering clinical and surgical patients together, the rate of appropriate prophylaxis (42.1%) was similar to a multicenter Brazilian study conducted in 2006 at five hospitals in the state of São Paulo, which reported a 42.7% overall rate of compliance with the prophylaxis protocol. 8

 Analyzing the outcome separately for clinical patients, prophylaxis was administered correctly in just 52.9% of cases, and even lower values were observed for surgical patients (37.5% of cases), reflecting rates slightly lower than found in the bibliographic review. 8 ^,^ 11 ^,^ 18 Analysis of data from Brazil collected in large-scale multicenter studies such as ENDORSE revealed rates of appropriate prophylaxis of 59% and 46% for clinical and surgical patients, respectively. The overall rates of appropriate prophylaxis in data from 32 countries were 40.0% for clinical patients and 59.0% for surgical patients, but there were major differences between different countries ( Table 10 ). 11

**Table 10:** Comparison with data collected in the ENDORSE multicenter study.

	**Present study** **n (%)**	**ENDORSE, Brazil data** **n (%)**	**ENDORSE: global data** **n (%)**
Surgical patients with appropriate prophylaxis	120 (37.5%)	192 (46.0%)	11.613 (59.0%)
Clinical patients with appropriate prophylaxis	72 (52.9%)	172 (59.0%)	61.119 (40.0%)

 Despite the well-known benefits of VTE prophylaxis, the persistently high rates of noncompliance with prophylaxis guidelines are disconcerting. The importance of the data observed emphasize the need to elucidate the reasons why appropriate prophylaxis rates are lower than those reported in the literature. Data were stratified with the objective of revealing where errors occur. Previous studies have not reported information on the reasons for noncompliance and the data observed in this study suggest that clinical and surgical patients are affected by different problems. 

 Among the clinical patients, the majority of noncompliance with guidelines was in the form of prescription of incorrect dosage/medication (26.4%), rather than failure to prescribe medication when indicated (20.5%). The opposite was the case with the surgical patients, 43.4% of whom were not given medication even though they met the criteria for it. Several factors may be behind the figures observed. 

 With relation to dose/medication errors, we observed that the majority of errors were prescription of the dosage recommended for high risk patients to moderate/low risk patients. In contrast, failure to prescribe pharmaceutical prophylaxis, which was most common among surgical patients, may be because of more than one reason: the cross-sectional nature of the study takes a snapshot at the time of prescription assessed (patients in the preoperative or immediate postoperative period may not yet have been medicated, in this case, correctly); surgeons may have been cautious with regard to risk of bleeding; the monthly rotation of the residents who work in the wards may be involved; risk stratification may have been incorrect; or the VTE prophylaxis protocol may not have been used or may not have been known about. A previous study conducted in Brazil with 105 physicians, surgeons, and clinicians attempted to define where VTE prophylaxis errors were being committed, administering a questionnaire on the subject to prescribers. It observed that 100% of the physicians knew the risk factors and the methods of VTE prophylaxis and that 92.3% of them knew how to use prophylaxis correctly. 19 ^,^ 20 Knowing that the care teams in the clinical/surgical wards are made up of residents who have passed rigorous examinations to be selected for residency programs and of specialist treating physicians, we do not think that ignorance of the protocol is the most plausible hypothesis for the low rates of appropriate prophylaxis. 

 Especially high rates of noncompliance with guidelines were observed for moderate risk surgical patients (40.9%), who were classified erroneously by prescribers who did not adhere to the SBACV guidelines. Many errors of non-prescription of prophylaxis originate from failure to perceive the magnitude of the problem and from individual experiences not founded in management of the subject. 21 ^,^ 22 Other services also commit prophylaxis errors and these could be corrected by maintaining ongoing education of prescribers and standardizing prescription according to risk protocols. The results of these interventions could raise appropriate prophylaxis rates from 43% to 71%. 3 Applications for smartphones and tablets, such as Caprini DVT Risk® and Thromboembolism Risk®, among others, facilitate risk stratification of patients in hospital and can be used free-of-charge, rapidly, and safely at the bedside. The medical residency programs at the institution studied include weekly lessons covering their respective specialties and subjects such as VTE prophylaxis and treatment should be emphasized in all disciplines that involve care for inpatients. Similar subjects could be dealt with for non-physician healthcare teams as part of programs for specialization and improvement. 

 Difficulties with and limitations restricting the medical record and prescription software used at the hospital became clear during analysis of the data. Currently, the prescription system warns the prescriber to make sure that the prophylactic medication really is indicated for patients when it is added to the prescription list. However, the system should work in the opposite manner, warning the physician to check whether the patient should be being prescribed pharmaceutical prophylaxis when this is not the case, or whether the prophylactic dose is correct for the patient’s risk class. The system could also contribute by alerting the physician if a patient has been admitted with cancer, pelvic fractures, or thrombophilias or to a stroke unit. The hospital studied does not have other types of heparin available for pharmaceutical prophylaxis or pneumatic boots for use on the wards, but use of specific elastic stockings and other types of low cost mechanical prophylaxis could easily be added, increasing prescription options. 

 The relevance of this study lies in the enormous social and economic cost that complications secondary to prophylaxis errors can cause. Venous thromboembolism is a silent and dangerous disease that is inherent to a large proportion of patients admitted to tertiary hospitals, but which is not always remembered by all treating physicians. 22

 When appropriately administered, VTE prophylaxis reduces morbidity and mortality and the cost of complications, admissions, and medications in all administrative areas, directly benefiting patients and the healthcare system. 18

## CONCLUSIONS

 At the University Hospital studied, the risk of VTE among inpatients was similar to rates reported in the literature. Prescriptions of VTE prophylaxis were correct in 42.1% of the patients in the entire sample and, when stratified, 52.9% of clinical patients and 37.5% of surgical patients received the correct VTE prophylaxis. The most common error causing incorrect prescription was failure to classify patient VTE risk. To reduce this problem, ongoing education is recommended for prescribers and non-prescribers, encouraging use of instruments for risk stratification and prescription compatible with risk levels, in addition to changes to the software used for medical records and electronic prescriptions to include risk assessment and prescription reminders for prevention of VTE. 
