# Supplementary material for: Venous thromboembolism, can we do better? Profile of venous thromboembolism risk and prophylaxis in a University Hospital in the State of São Paulo
Source: J Vasc Bras. 2019 Feb 27;18:e20180040. doi: 10.1590/1677-5449.004018 (PMC6579528; doi:10.1590/1677-5449.004018)
Supplement: Translation

# Translation: Profilaxia de tromboembolismo venoso, podemos fazer melhor? Perfil de risco e profilaxia de tromboembolismo venoso em Hospital Universitário do interior do Estado de São Paulo

**Authors:** Arthur Curtarelli, Luiz Paulo Correia e Silva, Paula Angeleli Bueno de Camargo, Rafael Elias Farres Pimenta, Rodrigo Gibin Jaldin, Matheus Bertanha, Marcone Lima Sobreira, Winston Bonetti Yoshida

**Affiliations:** 1 Universidade Estadual “Júlio de Mesquita Filho” – UNESP, Faculdade de Medicina de Botucatu (FMB), Hospital das Clínicas (HC), Botucatu, SP, Brasil.

**Keywords:** tromboembolia venosa, trombose venosa, embolia, trombose

## Abstract

**Contexto:**

O tromboembolismo venoso (TEV) é uma doença silenciosa e potencialmente letal que acomete parcela importante dos pacientes hospitalizados. Com alta morbimortalidade e elevado custo financeiro para o sistema de saúde, o TEV pode ser prevenido com uso da profilaxia, já estabelecida pela literatura. No mundo real, a profilaxia para TEV possui média de adequação inferior a 50%.

**Objetivos:**

Definir o perfil epidemiológico do doente com TEV em um hospital universitário e a taxa de adequação da profilaxia para TEV no referido serviço, além de determinar meios para melhorá-la.

**Métodos:**

Estudo transversal observacional realizado pela coleta de dados no prontuário médico dos pacientes que preencheram critérios de inclusão. Comparou-se a taxa de adequação da profilaxia para TEV prescrita para pacientes clínicos e cirúrgicos, segundo diretrizes da Sociedade Brasileira de Angiologia e Cirurgia Vascular (SBACV), de acordo com sua classificação de risco para TEV.

**Resultados:**

A taxa global de adequação das prescrições de profilaxia para TEV foi de 42,1% *versus* 57,9% de inadequação. Pacientes clínicos obtiveram taxa de adequação de 52,9%, enquanto pacientes cirúrgicos obtiveram taxa de adequação de 37,5%.

**Conclusões:**

As taxas de prescrição adequada para profilaxia para TEV ainda se encontram aquém do esperado. Educação continuada, estímulo à aplicação da estratificação de risco à beira do leito e adequações no sistema de prescrição eletrônica podem aumentar as taxas de prescrição adequada para profilaxia de TEV.

## INTRODUÇÃO

 As profilaxias medicamentosas e mecânicas do tromboembolismo venoso (TEV) estão bem estabelecidas em consensos internacionais, tanto em pacientes clínicos como cirúrgicos 1 ^,^ 2 , baseadas em modelos de estratificação de riscos 3 ^-^ 5 . No entanto, várias publicações nacionais 5 ^,^ 6 e internacionais mostram que, no mundo real, aproximadamente 50% dos pacientes em risco de TEV não estão recebendo profilaxia química quando indicada, ou a estão recebendo de forma inapropriada 7 ^,^ 8 . Os índices de adequação variam entre países e serviços desde 2% até 92% 9 ^-^ 11 . O Brasil possui, segundo o estudo ENDORSE, taxas de inadequação de 41% para doentes clínicos e 54% para doentes cirurgicos 11 . Estudos nacionais reiteram o achado, com taxas de inadequação ainda maiores: 61% para pacientes clínicos e cirúrgicos 12 . 

 Além de toda morbidade e mortalidade que uma internação complicada com TEV pode trazer – 2 milhões de casos de trombose venosa profunda (TVP) e 200 mil mortes/ano nos EUA –, os custos financeiros dessa doença têm preocupado administradores e gestores. Em um hospital universitário brasileiro, o custo de tratamento intra-hospitalar para TEV, até a obtenção de nível terapêutico, variou de US$ 69,11 quando tratado com heparina de baixo peso molecular (HBPM) a US$ 88,39 quando a opção foi heparina não fracionada (HNF), levando-se em conta apenas o valor do material e do medicamento utilizados, à exceção das bombas de infusão 13 . Valores ainda maiores podem ser atingidos para tratamento completo da doença em esquema de 90 dias em atendimento domiciliar: cifras de US$ 9.347,00 quando tratada com HBPM a US$ 11.930,00 quando tratada com HNF foram evidenciadas por uma coorte retrospectiva canadense 14 . 

 De acordo com as diretrizes nacionais e internacionais, a introdução de Comissões Hospitalares de Prevenção de TEV (CPTEV) seria um recurso importante para melhoria na prevenção de TEV 15 ^-^ 17 . Apesar de a profilaxia do TEV estar bem estabelecida através de consensos nacionais e internacionais, é desconcertante como sua inadequação ainda persiste nos centros médicos brasileiros. 

 As universidades estaduais do estado de São Paulo concentram os maiores e mais importantes hospitais universitários do interior do estado, responsáveis pelo atendimento de parcela expressiva da população que depende do Sistema Único de Saúde (SUS). A implantação de CPTEV vem sendo feita de forma tímida nessas instituições, e um levantamento da real situação da profilaxia do TEV precisa ser realizado de maneira extensa. 

 Os objetivos do presente estudo foram avaliar o perfil de risco de pacientes internados em um hospital público universitário brasileiro, verificar as taxas de inadequação da profilaxia para o TEV, identificar as causas dessas inadequações e sugerir meios para a resolução do problema. 

## MÉTODOS

 Trata-se de um estudo transversal observacional. Foram incluídos pacientes adultos com mais de 18 anos, internados em um hospital público universitário brasileiro e assistidos exclusivamente pelo SUS no período entre outubro de 2015 e fevereiro de 2016 nas especialidades de cirurgia ortopédica, cirurgia geral, gastrocirurgia, cirurgia vascular, urologia, ginecologia, clínica médica e terapia intensiva. Não foram incluídos os pacientes avaliados em internações prévias, gestantes, aqueles com contraindicação a anticoagulantes, com indicação de filtros de veia cava, em anticoagulação plena ou que não preencheram os critérios de inclusão acima. 

 Os dados foram coletados a partir de informações do prontuário eletrônico dos pacientes internados utilizando o software MV-PEP®, com autorização do Comitê de Ética e Pesquisa do hospital e com consentimento do médico responsável de cada especialidade, porém sem aviso prévio à equipe médica assistente. 

 O tamanho da amostra foi definido após avaliação estatística com dados preliminares de coleta piloto com informações de 80 pacientes. A amostra total foi calculada em 500 pacientes, com número similar de pacientes para cada especialidade. Os dados foram coletados de forma aleatória conforme as internações no período da coleta de dados. 

 Para avaliação do risco e indicação da profilaxia, foram utilizadas as diretrizes de profilaxia para TEV da Sociedade Brasileira de Angiologia e Cirurgia Vascular (SBACV) de 2005 ( Figura 1 ). Os dados foram coletados pelos pesquisadores e tabulados em planilha do software Excel® de forma padronizada para posterior análise estatística. 

**Figura 1:** Protocolo de profilaxia da trombose venosa profunda da Sociedade Brasileira de Angiologia e Cirurgia Vascular.

 Os pacientes foram divididos em dois grandes grupos (clínicos e cirúrgicos) e posteriormente subdivididos conforme a estratificação de risco para TEV da SBACV em clínicos de baixo e alto risco e cirúrgicos de baixo, moderado e alto risco 17 . Os resultados foram então confrontados por classificação de risco *versus* profilaxia prescrita, sendo consideradas adequadas somente aquelas previstas pelos critérios da diretriz de TEV da SBACV. Os dados e os resultados foram duplamente conferidos pelos pesquisadores. 

 Foram consideradas profilaxias mecânicas adequadas somente aquelas passiveis de prescrição no sistema de gerenciamento MV-PEP® do hospital: orientar/estimular deambulação precoce e fisioterapia motora. Outros métodos de profilaxia mecânica como uso de meias elásticas de compressão graduada e/ou de aparelhos de compressão pneumática intermitente não estavam à disposição da equipe médica assistente. Da mesma forma, foram consideradas adequadas profilaxias químicas somente aquelas previstas pela diretriz da SBACV e disponíveis no MV-PEP® do hospital: enoxaparina 20 mg uma vez ao dia, enoxaparina 40 mg uma vez ao dia, heparina não fracionada 5.000 unidades internacionais (UI) de 8/8 horas ou de 12/12 horas e fondaparinux 2,5 mg uma vez ao dia. As medicações daltaparina e nadroparina, também previstas pela SBACV, não estavam disponíveis no serviço. 

 A análise estatística foi realizada por meio do software *Statistical Package for the Social Sciences* (SPSS®), utilizando-se dos testes qui-quadrado para variáveis categóricas e teste *t* de Student para comparação entre médias de variáveis contínuas, contando com assessoria estatística do escritório de apoio à pesquisa da instituição. 

## RESULTADOS

### Elegibilidade e demografia

 Foram analisados os prontuários eletrônicos de 500 pacientes selecionados de forma aleatória. Destes, 456 (100%) preencheram critérios de inclusão, sendo subdivididos em dois grupos distintos: A) pacientes clínicos (n = 136, 29,8%) e B) pacientes cirúrgicos (n = 320, 70,2%), com base nos protocolos de risco e indicações de profilaxia da SBACV. Dos 44 pacientes excluídos da amostra, oito possuíam indicação para uso de filtro de veia cava, 16 eram gestantes e 20 estavam em anticoagulação plena ( Figura 2 ). 

**Figura 2:** Fluxograma de inclusões, exclusões e risco de tromboembolismo venoso para todos os pacientes.

 Os pacientes analisados permaneceram hospitalizados por motivos diversos e pelo tempo julgado necessário pela equipe médica assistente, sendo esta a responsável pela escolha e prescrição de profilaxia para TEV. 

 A predominância de pacientes do sexo masculino ocorreu tanto no grupo clínico (52,6% homens *versus* 47,4% mulheres) como no grupo cirúrgico (60,6% homens *versus* 39,4% mulheres). O grupo clínico teve média de idade superior à do grupo cirúrgico em 7,4 anos (62,4 *versus* 55,0 anos, respectivamente) e tempo de internação 1,5 dias mais longo se comparado ao do grupo de pacientes cirúrgicos (6,1 dias *versus*. 4,6 dias). O perfil demográfico dos pacientes encontra-se na Tabela 1 . 

**Tabela 1:** Fatores de risco para TEV por grupo, clínico e cirúrgico.

**Fator de risco**	**Clínico (%)**	**Cirúrgico (%)**
Idade média	62,4 anos	55,0 anos
Tempo de internação	6,1 dias	4,6 dias
Idade > 60 anos	59,2%	47,7%
Sepse/infecção grave	57,9%	16,1%
Sexo (feminino/masculino)	47,4%/52,6%	39,4%/60,6%
Internação em UTI	4,6%	6,9%
Cirurgia oncológica	3,9%	8,0%
Tempo cirúrgico > 60 minutos	3,9%	38,8%
IAM	3,9%	1,4%
Síndrome nefrótica	3,3%	0,3%
Idade entre 40-60 anos	28,3%	28,7%
Câncer	23,7%	14,1%
Restrito ao leito > 3 dias	23,7%	13,7%
ICC	20,4%	6,3%
Anticoncepcional oral	2%	0,6%
Paralisia de membro inferior	2,0%	1,1%
Acidente vascular cerebral	2,6%	0,0%
Anestesia geral	2,6%	24,1%
Varizes intensas	2,6%	5,5%
Cateter venoso de longa permanência	14,5%	12,1%
História previa de TEP/TVP	12,5%	6,6%
Quimioterapia	11,8%	3,4%
DPOC	11,2%	3,4%
Doença autoimune	1,3%	1,4%
Trauma	1,3%	19,0%
Trombofilia	0,7%	0,0%
Prostatectomia transvesical	0,7%	1,4%
Cirurgia ortopédica de quadril ou joelho	0,0%	4,0%
Trauma raquimedular	0,0%	9,0%
Queimadura extensa	0,0%	0,5%
Imobilização de membros	0,0%	21,3%
Retocolite ulcerativa	0,0%	0,6%
Terapia de reposição hormonal	0,0%	0,0%

TEP = tromboembolismo pulmonar, TVP = trombose venosa profunda, DPOC = doença pulmonar obstrutiva crônica, ICC = insuficiência cardíaca crônica, IAM = infarto agudo do miocárdio, UTI = unidade de terapia intensiva.

### Fatores de risco

 Assim como o perfil demográfico, os fatores de risco foram distintos em ambos os grupos. Houve superioridade de fatores de risco relacionados a doenças crônicas (câncer, doença pulmonar obstrutiva crônica, insuficiência cardíaca congestiva, infarto agudo do miocárdio, restrição em leito e história prévia de TEV) e medicações quimioterápicas no grupo clínico, enquanto que no grupo cirúrgico os fatores predisponentes para TEV foram relacionados à própria intervenção cirúrgica (trauma, lesão tissular, anestesia geral e imobilização pós-operatória) ( Tabela 1 ). 

### Desfecho global (grupos clínico e cirúrgico)

 Verificou-se taxa global de adequação para profilaxia TEV de 42,1% *versus* 57,9% de inadequação entre os 456 doentes analisados ( Tabela 2 ). 

**Tabela 2:** Análise da adequação e inadequação global do estudo (p < 0,05).

	**Clínico** **n (%)**	**Cirúrgico** **n (%)**	**% total**
Total	136 (29,8%)	320 (70,2%)	100%
Profilaxia adequada	72 (15,7%)	120 (26,3%)	42,1%
Profilaxia inadequada	64 (14,1%)	200 (43,8%)	57,9%

 A avaliação em separado dos 57,9% (264 pacientes) que tiveram profilaxia para TEV realizada de forma inadequada revelou que 36,6% (167 pacientes) preenchiam critérios para profilaxia química mas não a receberam; 14,4% (66 pacientes) a receberam com dose incorreta; 1,9% (nove pacientes) a receberam com intervalo posológico incorreto; e 4,8% (22 pacientes) receberam medicamento mesmo não preenchendo critérios para profilaxia química ( Tabela 3 e Figura 3 ). 

**Tabela 3:** Análise estratificada das formas de inadequação da profilaxia entre todos os pacientes com profilaxia inadequada (p < 0,05).

**Pacientes com profilaxia inadequada**		**n (%) global**	**n (%) global**
Não receberam profilaxia química			167 (36,6%)
Receberam profilaxia inadequada	Receberam dose inadequada	66 (14,4%)	97 (21,2%)
	Receberam intervalo posológico inadequado	9 (1,9%)	
	Não preenchiam critério, porém receberam profilaxia química	22 (4,8%)	
	Receberam medicamento inadequado	0 (0,0%)	

**Figura 3:** Fluxograma de inclusões, exclusões, adequação, inadequação e suas subformas para todos os pacientes.

### Desfecho do grupo clínico

 A análise em separado dos 136 (100%) pacientes clínicos revelou que 72 (52,9%) deles receberam profilaxia química da maneira adequada, conforme orientação da diretriz da SBACV, enquanto que 64 (47,1%) pacientes tiveram profilaxia química realizada de forma inadequada ( Tabela 4 ). 

**Tabela 4:** Análise da adequação e inadequação total dos doentes clínicos.

**Pacientes clínicos**	**n total**	**% total**
Profilaxia adequada	72	52,9%
Profilaxia inadequada	64	47,1%

 A SBACV classifica os pacientes clínicos em doentes de risco aumentado, que devem receber a profilaxia química, e doentes que têm risco diminuído e não devem receber medicamentos profiláticos, sendo tratados apenas com profilaxia mecânica. Seguindo a orientação da SBACV, esses pacientes foram assim classificados e analisados separadamente sobre a adequação ou não de sua profilaxia química. 

 Do total de 112 pacientes clínicos com risco aumentado, a profilaxia foi prescrita adequadamente (ou seja, indicou-se profilaxia química) em 60 pacientes (44,1% dos pacientes clínicos), e inadequadamente nos demais pacientes (38,2% dos pacientes clínicos) ( Tabela 5 ). 

**Tabela 5:** Análise estratificada de pacientes clínicos pela necessidade ou não de profilaxia e sua adequação ou inadequação (p < 0,05).

**Risco para TVP/TEP**	**Pacientes clínicos** **n (%)**	**Profilaxia inadequada** **n (%)**	**Profilaxia adequada** **n (%)**
Devem receber profilaxia química	112 (82,3%)	52 (38,2%)	60 (44,1%)
Não devem receber profilaxia química	24 (17,7%)	12 (8,8%)	12 (8,8%)

 Ao observar somente o conjunto de doentes clínicos com profilaxia química inadequada (64 doentes) observamos que 28 (20,5% dos pacientes clínicos) pacientes não receberam o medicamento quando preenchiam critério para tal, enquanto os outros 36 (26,4% dos pacientes clínicos) pacientes possuíam prescrição de profilaxia química, porém em desencontro ao protocolo da SBACV por dose inadequada ou intervalo posológico incorreto, ou receberam medicação mesmo não preenchendo critérios para profilaxia química ( Tabela 6 e Figura 4 ). 

**Tabela 6:** Análise estratificada das formas de inadequação da profilaxia entre pacientes clínicos com profilaxia inadequada (p < 0,05).

**Pacientes clínicos com profilaxia inadequada**		**n (%) global**	**n (%) global**	**% entre os inadequados**
Não receberam profilaxia química			28 (20,5%)	43,8%
Receberam profilaxia inadequada	Receberam dose inadequada	18 (13,2%)	36 (26,4%)	56,2%
	Receberam intervalo posológico inadequado	6 (4,4%)		
	Não preenchiam critério, porém receberam profilaxia química	12 (8,8%)		
	Receberam medicamento inadequado	0 (0,0%)		

**Figura 4:** Fluxograma de inclusões, exclusões, adequação, inadequação e suas subformas para pacientes clínicos.

### Desfecho do grupo cirúrgico

 A avaliação em separado do grupo de pacientes cirúrgicos, com 320 (100%) doentes, teve taxa de adequação de 37,5% (120 pacientes) e inadequação de 62,5% (200 pacientes) segundo a diretriz da SBACV ( Tabela 7 ). 

**Tabela 7:** Análise da adequação e inadequação total dos doentes cirúrgicos.

**Pacientes cirúrgicos**	**n total**	**% total**
Profilaxia adequada	120	37,5%
Profilaxia inadequada	200	62,5%

 Pacientes cirúrgicos são, segundo a diretriz da SBACV, subdivididos em três categorias: baixo, moderado e alto risco, cada qual com sua forma adequada de realização da profilaxia química. 

 Observou-se que 37,5% pacientes cirúrgicos receberam profilaxia adequada, distribuídos da seguinte forma, considerando sua categoria de risco: 14,4% eram de baixo risco para TVP/TEP, 5,3% eram de moderado risco, e 17,8% eram de alto risco ( Tabela 8 ). 

**Tabela 8:** Análise estratificada de pacientes cirúrgicos pela classificação do risco e adequação ou não da profilaxia para TEV (p < 0,05).

**Risco para TVP/TEP**	**Pacientes cirúrgicos** **n (%)**	**Profilaxia inadequada** **n (%)**	**Profilaxia adequada** **n (%)**
Alto risco	116 (36,2%)	59 (18,4%)	57 (17,8%)
Moderado risco	148 (46,2%)	131 (40,9%)	17 (5,3%)
Baixo risco	56 (17,5%)	10 (3,1%)	46 (14,4%)

 Entre os 200 doentes com esquema profilático divergente do recomendado pela diretriz da SBACV, 139 (43,4%) pacientes não receberam profilaxia química quando preenchiam critérios para uso do medicamento, ao passo que os outros 61 (19,0%) pacientes receberam profilaxia química, porém com dose ou posologia inadequada, ou receberam medicação mesmo não preenchendo critérios para profilaxia química ( Tabela 9 e Figura 5 ). 

**Tabela 9:** Análise estratificada das formas de inadequação da profilaxia dentre pacientes cirúrgicos com profilaxia inadequada (p < 0,05).

**Pacientes cirúrgicos com profilaxia inadequada**		**n (%) global**	**n (%) global**	**n (%) global**	**n (%) global**
Não receberam profilaxia química					139 (43,4%)
Receberam profilaxia inadequada		Baixo risco	Moderado risco	Alto risco	61 (19,0%)
	Receberam dose inadequada	0 (0,0%)	32 (10,0%)	16 (5,0%)	
	Receberam intervalo posológico inadequado	0 (0,0%)	0 (0,0%)	0 (0,0%)	
	Não preenchiam critério, porém receberam profilaxia química	10 (3,1%)	0 (0,0%)	0 (0,0%)	
	Receberam medicamento inadequado	0 (0,0%)	0 (0,0%)	0 (0,0%)	

**Figura 5:** Fluxograma de inclusões, exclusões, adequação, inadequação e suas subformas para pacientes cirúrgicos.

## DISCUSSÃO

 Os dados coletados reafirmam o perfil epidemiológico descrito na literatura para pacientes com risco aumentado para TEV, visto que encontramos pacientes com múltiplas comorbidades, idade superior a 60 anos e tempo de internação médio de 4,6 a 6,1 dias. Os pacientes cirúrgicos parecem possuir ainda mais riscos, em decorrência de fatores diretamente ligados ao procedimento cirúrgico. 

 Considerando os doentes clínicos e cirúrgicos em conjunto, a taxa de adequação (42,1%) foi semelhante à encontrada em estudo nacional multicêntrico realizado em 2006 em cinco hospitais do estado de São Paulo, que evidenciava 42,7% de adequação global ao protocolo de profilaxia 8 . 

 No desfecho em separado dos pacientes clínicos, a profilaxia foi feita adequadamente em apenas 52,9% dos casos, e valores ainda menores foram observados em doentes cirúrgicos (37,5% dos casos), refletindo taxas ligeiramente inferiores às observadas na revisão bibliográfica realizada 8 ^,^ 11 ^,^ 18 . Grandes estudos multicêntricos como o ENDORSE revelaram, na avaliação em separado com dados do Brasil, taxas de adequação de 59% e 46% para pacientes clínicos e cirúrgicos, respectivamente. A taxa global de adequação, envolvendo dados de 32 países, foi de 40,0% em doentes clínicos a 59,0% em doentes cirúrgicos, com grandes diferenças entre as nações 11 ( Tabela 10 ). 

**Tabela 10:** Comparação dos dados coletados com estudo multicêntrico ENDORSE.

	**Presente estudo** **n (%)**	**ENDORSE dados Brasil** **n (%)**	**ENDORSE dados globais** **n (%)**
Pacientes cirúrgicos com profilaxia adequada	120 (37,5%)	192 (46,0%)	11.613 (59,0%)
Pacientes clínicos com profilaxia adequada	72 (52,9%)	172 (59,0%)	61.119 (40,0%)

 A despeito dos conhecidos benefícios da profilaxia de TEV, é desconcertante como persistem altas taxas de inadequação à diretriz de profilaxia. A importância dos dados encontrados reforça a necessidade de esclarecimento sobre motivo de as profilaxias terem índices de adequação abaixo dos relatados na literatura. A estratificação dos dados teve como objetivo esclarecer onde incorreram esses erros. Estudos anteriores não trouxeram informações quanto aos motivos das inadequações, e os dados do presente estudo sugerem problemas diferentes para pacientes clínicos e cirúrgicos. 

 As inadequações dos pacientes clínicos ocorreram em sua maioria pela prescrição de dose/medicamento inadequado (26,4%) e não pela não prescrição da medicação quando indicada (20,5%). O oposto ocorreu com doentes cirúrgicos, dos quais 43,4% não receberam o medicamento mesmo preenchendo critérios para uso. Diversos fatores podem justificar os valores encontrados. 

 Nas inadequações por dose/medicamento, observamos que o erro aconteceu principalmente pela prescrição a doentes de moderado/baixo risco da dose adequada a doentes de alto risco. Em contrapartida, a não prescrição da profilaxia química, mais evidente em doentes cirúrgicos, pode ser explicada por mais de um motivo: a transversalidade do estudo foi pontual no momento da prescrição avaliada (doentes em pré-operatório ou pós-operatório imediato podem não ter recebido a medicação, neste caso de forma correta); cautela do cirurgião quanto ao risco de sangramento; rodízio mensal dos médicos residentes que assistem as enfermarias; estratificação de risco incorreta; não aplicação ou desconhecimento do protocolo de profilaxia para TEV. Em estudo anterior feito no Brasil com 105 médicos, cirurgiões e clínicos, tentou-se definir onde se incorriam os erros na profilaxia para TEV submetendo os prescritores a um questionário sobre o tema. Observou-se que 100% dos médicos conheciam os fatores de risco, bem como as formas de profilaxia para TEV, e que 92,3% deles sabiam utilizar a profilaxia de maneira adequada 19 ^,^ 20 . Tendo conhecimento de que a equipe assistente das enfermarias clínicas/cirúrgicas é formada por médicos residentes que foram submetidos a criteriosa prova para os programas de residência e por médicos assistentes especialistas, acreditamos que o desconhecimento do protocolo não seja a hipótese mais plausível para as baixas taxas de adequação. 

 Taxas especialmente altas de inadequação foram observadas em doentes cirúrgicos de moderado risco (40,9%), os quais foram classificados de forma errônea pelos prescritores que não se atentaram para a diretriz da SBACV. Muitos erros de não prescrição da profilaxia vieram da não percepção da magnitude do problema e de experiências individuais não embasadas no manejo do tema 21 ^,^ 22 . Outros serviços também cometem erros de profilaxia, sendo que essas inadequações podem ser corrigidas ao se manter educação continuada aos prescritores e padronizações de prescrição segundo protocolos de risco. Os resultados dessas intervenções podem elevar as taxas de adequação de 43% para 71% 3 . Aplicativos para *smartphones* e *tablets*, como o Caprini DVT Risk® e Tromboembolism Risk®, entre outros, facilitam a estratificação de risco dos pacientes internados e podem ser utilizados de forma gratuita, rápida e segura à beira do leito. Os programas de residência médica da instituição em estudo contemplam, em aulas semanais, suas diversas especialidades, e temas como profilaxia e tratamento de TEV deveriam ser ressaltados por todas as disciplinas que envolvem a assistência a pacientes em leito de internação. Tópicos semelhantes podem ser abordados para equipes não médicas em programas de especialização e aprimoramento. 

 Dificuldades e limitações relacionadas ao *software* de prontuário e prescrição de pacientes utilizado pelo serviço tornaram-se nítidas após a análise dos dados. Atualmente, o sistema de prescrição alerta o prescritor a observar se o paciente realmente possui indicação do fármaco profilático quando ele é adicionado à lista de prescrição. No entanto, o sistema deveria trabalhar de forma inversa, alertando o médico a observar se o paciente não deveria receber a profilaxia química quando esta não foi prescrita, ou ainda se a dose da profilaxia estaria adequada segundo a estratificação de risco do paciente. O sistema poderia contribuir também alertando o médico quando o paciente for internado com diagnóstico de doença oncológica, fraturas da bacia, trombofilias, ou em unidade de acidente vascular cerebral. O hospital em questão não possui disponibilidade de outras formas de heparina para profilaxia química ou bota pneumática para uso em enfermaria, porém o uso de meia elástica especifica e outras formas de profilaxia mecânica de baixo custo poderiam ser facilmente acrescentados, incrementando as possibilidades de prescrição. 

 A relevância deste estudo encontra-se no enorme custo social e econômico que as complicações secundárias aos erros na profilaxia podem desencadear. O TEV é uma doença silenciosa e perigosa, inerente à grande parcela de pacientes admitidos em hospital terciário e que nem sempre está na lembrança de todos os médicos assistentes 22 . 

 Realizada de maneira adequada, a profilaxia para TEV diminui morbimortalidade, gastos com complicações, internações e medicações em todas as esferas administrativas, trazendo benefício direto para os doentes e para o sistema de saúde 18 . 

## CONCLUSÕES

 No hospital universitário em estudo, o risco de TEV em pacientes internados foi similar ao estabelecido na literatura. A prescrição de profilaxia para TEV foi adequada em 42,1% dos pacientes de forma global e, quando estratificada, foi de 52,9% para doentes clínicos e 37,5% para pacientes cirúrgicos. O erro mais frequente que levou à prescrição incorreta foi a não estratificação do risco de TEV do paciente. Para redução desse problema, recomenda-se educação continuada para prescritores e não prescritores, com estímulo ao uso de instrumentos de estratificação de risco e de prescrição adequada a ela, além de modificações no programa de prontuário e prescrição eletrônica de modo a se incluir alertas de avaliação e de prescrição para prevenção do TEV.
